# Potential Therapeutic Appliances of Dietary Polyphenols: Resveratrol and Curcumin in Treatment of Gliomas

**DOI:** 10.3390/ijms26136154

**Published:** 2025-06-26

**Authors:** Ewa Smolińska, Mikołaj Grabarczyk, Weronika Justyńska, Aleksandra Bielenin, Andrzej Glabinski, Piotr Szpakowski

**Affiliations:** 1 Medical Faculty, Medical University of Lodz, 90-419 Lodz, Poland; ewa.smolinska@stud.umed.lodz.pl (E.S.); mikolaj.grabarczyk@stud.umed.lodz.pl (M.G.); weronika.justynska@stud.umed.lodz.pl (W.J.); aleksandra.bielenin@stud.umed.lodz.pl (A.B.); 2 Department of Neurology and Stroke, Medical University of Lodz, Zeromskiego 113 Street, 90-549 Lodz, Poland; andrzej.glabinski@umed.lodz.pl

**Keywords:** polyphenols, resveratrol, curcumin, gliomas, glioblastoma

## Abstract

Plant-derived polyphenols have become a subject of scientific interest in recent decades due to their widespread occurrence in dietary sources and multi-faceted biological activity, with many of these compounds being recognized as antioxidants and anti-inflammatory agents. Several of these chemicals have, moreover, attracted further interest as their anti-tumoral capabilities were discovered, promising potential implementation in the treatment of proliferative diseases, including various cancers. Malignancies of the central nervous system, the most prevalent of which are glioblastomas, are noted for their aggressiveness, dismal prognosis and low survival rates. This review focuses on two polyphenols with the most expansive body of research on this topic, namely resveratrol and curcumin. It covers recent developments in the research, including in vitro findings, animal model studies and clinical trials on these compounds’ effects on the growth and progression of glial tumors of the central nervous system. Its aim is to present the latest findings on the subject of the mechanisms of action of these phytochemicals and their synergistic activity with conventional therapies, as well as strategies to improve their efficacy for future therapeutic applications.

## 1. Introduction

Polyphenols are a diverse group of chemicals produced by plants as a defensive strategy against damaging agents, such as microbes and ultraviolet radiation, exhibiting various mechanisms of action and pleiotropic effects. Many of them have been identified in common dietary sources or plants which have long been utilized in traditional medicine. Over the past three decades, they have become a subject of scientific interest due to their antioxidant, anti-inflammatory, anti-oncogenic and neuroprotective effects, with hopes of therapeutic application in human diseases [1,2,3]. Polyphenols are defined chemically as compounds containing at least one aromatic nucleus and at least one –OH group. Based on their chemical structure, polyphenols are divided into three main groups—phenolic acids, stilbenes and flavonoids—the latter of which are further subdivided into subgroups including flavonols, flavones and others. Over 8000 such compounds have been identified so far [4,5,6]. This review focuses on two polyphenols, namely resveratrol and curcumin, which have been studied for their possible effects on glial tumors of the central nervous system. Numerous in vitro and in vivo studies have been conducted in order to elucidate their mechanisms of action and to search for ways of improving their pharmacokinetic properties. Resveratrol (3,5,4′-trihydroxy-trans-stilbene) is a derivative of stilbene found in blueberries, mulberries, cranberries and peanuts, as well as the skin of grapes and their derived product, wine. It first became a subject of widespread scientific interest in 1992, when it was suspected to improve cardiovascular health—an effect which has been dubbed the “French paradox” and attributed to red wine consumption [7,8]. Although this effect has since been questioned, other properties of this polyphenol have come to light. In 1997, it was discovered by Jang et al. that resveratrol exhibited activity against the three stages of tumorigenesis, affecting tumor initiation, promotion and progression in vitro [9]. Since then, knowledge about resveratrol’s anti-tumoral properties has developed, revealing the chemical’s ability to halt the proliferation of various neoplasms, including lymphoid and myeloid leukemia, colon, stomach and thyroid cancers, and melanoma [10]. Of interest to this review is the compound’s anti-tumoral activity against gliomas, which has been observed in vitro as well as in murine models. Resveratrol has been reported to induce cell cycle arrest and oxidative damage in glioma cells as well as enhance the anti-glioma efficacy of the chemotherapeutic drug temozolomide (TMZ) [11]. Curcumin ((1E,6E)-1,7-Bis(4-hydroxy-3-methoxyphenyl)hepta-1,6-diene-3,5-dione) is a phenolic compound found in turmeric, a spice obtained from the rhizomes of *Curcuma longa* and used in cooking [12]. It has been studied for its properties as a reactive oxygen species (ROS) scavenger and anti-inflammatory agent in vitro, although low bioavailability limits its application in vivo [13]. Nevertheless, some studies on humans have been conducted that suggest its potential role in the treatment of inflammatory diseases, such as rheumatoid arthritis and ulcerative colitis [14,15]. Several clinical trials are in progress to examine its effects in patients with various cancers, including breast, lung, prostate and colorectal cancer [16]. In tumors of the nervous system, curcumin has been studied in vitro for its pro-apoptotic properties and ability to sensitize glioma cells to radiation—qualities which have also been explored in in vivo studies on murine models. Curcumin has been employed in trials on human glioma patients: one inconclusive 2016 study offered cautiously optimistic results, and another study exploring its synergism with TMZ is in progress as of 2025 [17,18].

## 2. Gliomas: Overview

Tumors of the central nervous system have proved to be some of the most challenging neoplasms to treat. According to the GLOBOCAN report, 308,000 cases of brain tumors were diagnosed in 2020, with over 250,000 deaths in the same year, placing them among the more deadly cancers [19]. The proximity to vital structures in the brain as well as the need for aggressive therapy lead to a worsened quality of life, while the prognosis and survival rate remain dismal. Among primary tumors of the brain, the most prevalent are gliomas, which develop from glial or precursor cells and encompass a variety of histological subtypes. Of those, the most common is glioblastoma (GBM), which accounts for over 50% of gliomas. With an incidence of 3.2 per 100,000, it is also the most common malignant primary tumor of the brain in general. The survival estimate is very low, with a five-year survival rate calculated at 5.1%. Its high prevalence and aggressiveness make it a significant clinical challenge and the subject of extensive research, which is why the use of polyphenols in glioblastoma specifically will be the chief focus of this review [20,21]. GBM is classified as a grade IV glioma, more commonly diagnosed in older adults, with a median age of 64 and the highest incidence rates between the ages of 75 and 84, although it is not unheard of in younger adults and pediatric populations. GBMs occur more frequently in patients with various hereditary syndromes, such as Li Fraumeni syndrome and neurofibromatosis, although most cases of GBM are sporadic [21]. In the 2016 WHO classification, GBMs are divided based on the mutational status of the isocitrate dehydrogenase (IDH) gene, which is a vital prognostic factor. IDH-mutant glioblastomas exhibit better outcomes than IDH-wildtype glioblastomas. Another prominent marker is the methylated-DNA-protein-cysteine methyltransferase (MGMT) protein, a DNA repair enzyme whose increased levels in GBM cells have been associated with worse prognosis. Other genomic and molecular abnormalities have been observed in glioblastomas as well, including epidermal growth factor receptor (EGFR) amplification and TP53 mutations [22]. The clinical presentation of GBM is varied and dependent on the location of the tumor, with focal symptoms such as hemiparesis and sensory malfunction being relatively frequent. Non-specific symptoms such as headache, cognitive impairment and gait imbalance may present diagnostic difficulty, as they are often mistaken for signs of other disorders which are common in the elderly. As any intracranial mass does, GBMs pose a risk of high intracranial pressure and edema, which may be life-threatening [20,23]. GBMs are usually treated with a combination of surgery, radiotherapy and systemic chemotherapy, involving alkylating agents such as TMZ as well as monoclonal antibodies, including bevacizumab. Despite these efforts, GBM therapy is still unsatisfactory, with poor outcomes and significantly diminished quality of life [20]. Beneficial effects of plant-derived polyphenols in brain tumors, including gliomas, have been observed in vitro for over two decades now, promising possible new therapeutic options. These compounds offer a hope of enhancing the efficacy of standard chemotherapy, allowing clinicians to decrease its dosage and alleviate side effects, and ultimately to improve the dismal prognosis for GBM patients. However, none of the compounds discussed here have been successfully translated into clinical use due to their poor bioavailability. Much of the research is focused on elucidating the exact mechanisms responsible for the polyphenols’ anti-tumoral properties, as well as on improving the pharmacokinetic properties of these compounds, in hope of eventually employing them as novel therapeutic agents.

## 3. Resveratrol’s Role in Treatment of Gliomas

### 3.1. Reports from In Vitro Studies on Resveratrol Activity

**Figure 1:**
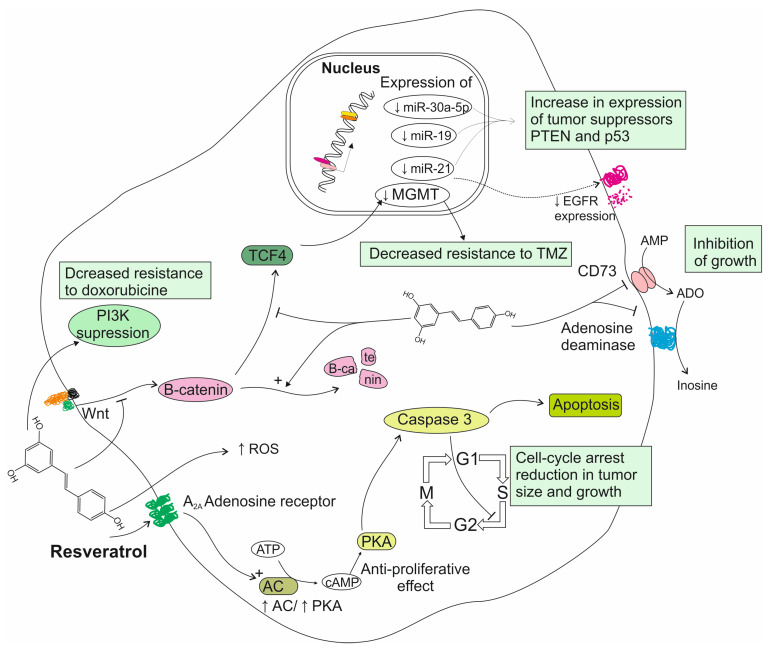
The impact and mechanisms of action of resveratrol on the biology of glioma cells.

Since the anti-tumoral potential of resveratrol was first reported by Jang et al. in 1997, the compound’s anti-proliferative properties have been investigated in several types of cancer [9]. Regarding malignancies of the nervous system, the research has been chiefly focused on glioma, promising potential therapeutic options which could improve the tumor’s poor prognosis. Zhang et al. demonstrated that resveratrol was able to induce cell cycle arrest in rat C6 glioma cells, noting an elevated number of cells in the S phase as well as an increase in cellular apoptosis, with caspase-3 acting as a mediator [24]. The exact mechanisms responsible for this anti-tumoral effect are not yet fully understood, with a few possible explanations having been explored in in vitro studies. Wang et al. found that resveratrol inhibited the expression of oncogenic microRNAs (miRs), which are overexpressed in glioma cell lines and have been shown to promote glioma formation. The expression of miR-21, miR-19 and miR-30a-5p in glioma cells was downregulated after resveratrol treatment, leading to the increased expression of the tumor-suppressor PTEN and p53 genes. Potentially due to the inhibition of miR-21, resveratrol also reduced the overexpression of EGFR, which has been shown to be an important anti-apoptotic and oncogenic factor in gliomas [25]. Studies conducted by Sánchez-Melgar et al. suggest that the anti-proliferative effect of resveratrol on glioma cells may be partially mediated by adenosinergic signaling. It has been found that resveratrol acts as a non-selective ligand for adenosine receptors in C6 glioma cells, consequently modulating the adenylyl cyclase (AC)/protein kinase A (PKA) pathway. In another study, it was observed that resveratrol-treated glioma cells presented diminished viability, increased apoptosis and cell cycle arrest in the G1 phase, as well as reduced levels of enzymatic activity of CD73 and adenosine deaminase (ADA), two enzymes involved in adenosine synthesis and metabolism. Since high CD73 activity has been observed in gliomas and glioblastomas and is suspected to promote tumor growth, the inhibition of CD73 activity may be partially responsible for the anti-tumoral properties of resveratrol. Cell cycle arrest is attributed to the activation of caspase 3 as a result of the binding of resveratrol molecules to adenosine receptors and the modulatory effect of these receptors on other signaling pathways. Given the multi-faceted downstream results of the binding of resveratrol to adenosine receptors, the authors emphasize the potential use of these receptors as targets for therapeutic interventions [26,27]. Other studies also note a marked increase in ROS levels after the resveratrol treatment of glioblastoma cells, suggesting that oxidative stress may be another mechanism responsible for the compound’s cytotoxicity, as has been found in studies on other tumors (incl. ovarian, colon and lung cancers) [28]. Other recent studies have explored the potential therapeutic use of resveratrol as a chemo-sensitizing agent in GBM treatment. The increasing resistance to TMZ, the current gold standard of GBM chemotherapy, has proved to be a significant clinical challenge in the treatment of this tumor. It was found that resveratrol enhances TMZ efficacy in low-sensitivity GBM cells through the inhibition of the Wnt/B-catenin signaling pathway and the downregulation of MGMT, a DNA repair protein which has been suspected to be partially responsible for TMZ resistance [29]. In agreement, studies by Liu et al. and Wu et al. found that combined TMZ + resveratrol treatment resulted in lower MGMT levels in GBM cells, additionally noting the inhibition of the STAT3 signaling pathway, whose downstream effects contribute to cancer progression. It is proposed that a combination strategy for TMZ + resveratrol treatment should be developed, as decreased TMZ resistance would allow for the use of lower TMZ doses, thus lowering the risk of side effects [30,31]. In another study it was found that resveratrol was able to overcome doxorubicin resistance in GBM cells by upregulating PTEN and suppressing the PI3K pathway [32]. These findings offer promise for a new therapeutic approach, utilizing this polyphenol in combination with TMZ or doxorubicin to improve the response to chemotherapy in GBM patients. Some studies have found that resveratrol enhanced the sensitivity of cancer cells to radiation. Yang et al. attributed this effect to the inhibition of STAT3 signaling in GBM tumor-initiating cells. Resveratrol suppresses the increase in STAT3 levels which is observed in irradiated cells and leads to their resistance to radiation. As post-surgical radiotherapy is one of the standard forms of treatment in glioblastoma, resveratrol could be utilized as an adjuvant drug in overcoming radiation resistance and improving treatment effectiveness [33,34]. The modes of resveratrol action on glioma cells are summarized in Figure 1.

### 3.2. Reports from Animal Models on Resveratrol Activity

Resveratrol has been found to reduce cell growth and induce apoptosis in glioma-bearing murine models in a time- and dose-dependent fashion, yet it presents the challenges of low bioavailability and low concentration in the brain, hindering its translation to clinical use [35,36,37]. It has been noted that the drug’s efficacy was significantly lower in rats with intracerebral gliomas when compared to rats who had been inoculated subcutaneously with glioma cells [37]. Resveratrol was detectable but did not reach therapeutic levels in rats’ brains when administered by conventional systemic administration routes, with lumbar puncture proving the most effective in ensuring high drug concentration at the site of the tumor [35]. In agreement, Clark et al. found that orally administered resveratrol in mice was able to suppress glioblastoma growth, but did not cause tumor regression, in contrast to direct intracranial administration, which greatly increased resveratrol concentration and efficacy [36]. These findings suggest resveratrol’s ability to cross the blood–brain barrier (BBB); however, methods of improving the compound’s availability should be developed. Figueiro et al. explored the efficacy of resveratrol enclosed in lipid-core nanocapsules (LNCs) in comparison to free resveratrol in a rat glioma model. Both compounds were administered intraperitoneally to rats. It was found that resveratrol-saturated LNCs had an anti-tumoral effect at doses as low as 5 mg/kg/day, whereas the free polyphenol showed no significant results at this dose. Interestingly, in the same study it was observed that resveratrol LNCs exhibited a higher degree of selectiveness for glioma cells and an absence of toxicity for typical neural tissue in vitro [38]. Transferrin receptors, which have been found to be overexpressed in various cancers, including gliomas, have been investigated as a possible target for increasing drug specificity and efficacy. It was found that resveratrol conjugated with polylactide-polyethylene glycol (PEG-PLA) particles and modified with transferrin was more effective in reducing glioma volume than free resveratrol in rats via intraperitoneal administration. This result was attributed to transferrin-mediated transcytosis across the BBB [39]. Jhaveri et al. demonstrated that resveratrol-loaded liposomes were more effective than free resveratrol in inducing apoptosis and reducing tumor size in GBM-bearing nude mice models. Drug efficacy and animal survival rate were further enhanced by modifying the liposomes with transferrin. Although none of the particles studied were able to successfully stop tumor growth, the authors suggest that their effect may be improved by increasing dosage or reducing particle size to ~100 nm [40]. Yi et al. have found that elastin-like polypeptide (ELP) conjugated with interferon (administered intra-tumorally) and resveratrol (administered intraperitoneally) demonstrated significant synergistic effects and successfully reduced glioblastoma growth [41]. Penalva et al. found that encasing resveratrol in casein nanoparticles increased the oral bioavailability of resveratrol tenfold. Compared to a free solution of the polyphenol, casein-encased resveratrol resulted in sustained levels of resveratrol in plasma for 8 h post-administration [42]. Ha et al. have demonstrated that pure trans-resveratrol nanoparticles (without any sugar or polymer additives) demonstrate a higher oral bioavailability in rats when prepared using the supercritical antisolvent process involving alcohol and dichloromethane mixtures [43]. In agreement with in vitro findings, resveratrol has proved effective as a chemo-sensitizing agent for TMZ glioma therapy in animal-model-based studies [29,44]. Xu et al. developed a co-loaded drug system using methoxypolyethylene glycol-caprolactone (mPEG-PCL) nanoparticles loaded with resveratrol and TMZ. This co-encapsulation preserved the synergistic effect of the two drugs and successfully increased cytotoxicity when compared to the free compounds [45]. Results from studies investigating the effect of resveratrol on gliomas in vivo studies are summarized in Table 1. The findings of these studies are promising, although limitations such as small sample size, differences in dosage and administration routes, as well as differences between murine and human biology, must be taken into account. Further in vitro and in vivo research is advisable to determine efficacious and safe drug delivery systems before resveratrol can be employed in clinical trials for glioblastoma treatment.

## 4. Curcumin’s Role in Treatment of Gliomas

### 4.1. Reports from In Vitro Studies on Curcumin Activity

Curcumin has been observed to exhibit anti-tumoral properties in various cancers in vitro. This effect has been reported to be p53- and caspase-independent, which allows curcumin to bypass common mechanisms of chemoresistance in glioblastoma cells. Instead, it has been observed that curcumin decreased the activity of transcription factors in GBM cells, including activator protein 1 (AP-1) and nuclear factor kappa-β (NF-κβ). Curcumin has been found to decrease GBM chemoresistance to standard chemotherapeutics, including doxorubicin, cisplatin, etoposide and paclitaxel, showing a marked increase in cytotoxicity in combined treatment. This chemo-sensitizing effect correlates with the downregulation of the anti-apoptotic proteins of the NF-κβ-regulated Bcl family, as well as the decreased expression of DNA repair enzymes, including MGMT [46,47,48,49]. It has been recently found that curcumin demonstrated a synergistic anti-proliferative and apoptotic effect on glioma cells with the plant-derived compound thymoquinone, which has also been studied for its potential therapeutic use [50]. Curcumin was also found to cause autophagy in glioma cells via the inhibition of the Akt/mTOR pathway and the activation of the ERK1/2 pathway, as well as through inducing cell cycle arrest in the G2/M phase, resulting in a significant reduction in tumor size and growth [51]. More recent studies conducted on GBM cells revealed that curcumin inactivated the ubiquitin ligase NEDD4, which has been suspected to play a role in tumorigenesis by promoting cell proliferation via the PI3K/Akt pathway [52]. It has also been observed that curcumin was able to induce apoptosis in GBM cells via the generation of ROS [53,54]. The different modes of curcumin activity on glioma tumors are summarized in Figure 1. These effects have made curcumin an attractive subject of research, considering its potential use in glioma therapy. Despite these promising findings, translation into clinical use has proved challenging due to curcumin’s poor oral bioavailability, hydrophobic properties and difficulty crossing the BBB. In recent years, research has been largely focused on investigating methods of improving the compound’s physicochemical characteristics and its use as a chemo-sensitizing agent in combined treatment for GBM. Some studies have attempted to develop curcumin delivery methods using nanoparticulate technologies. Cui et al. investigated a magnetic, transferrin-targeted polylactic-co-glycolic acid (PLGA) nanoparticulate dual-drug delivery system involving curcumin and paclitaxel, which resulted in increased uptake by glioma cells and an increased synergistic effect of the two drugs compared to non-targeted systems [55]. In another study, Rahman et al. studied a dual-drug system using lipid nanocapsules containing curcumin and paclitaxel. They observed an increased cytotoxic effect of the drug combination on GBM cells in comparison to nanocapsules containing each of the drugs delivered separately [56]. Piwowarczyk et al. found that various phytocompounds, including curcumin, encased in liposomal nanoformulations exhibited stronger cytotoxic effects against GBM cells than the free compounds. The authors additionally note that curcumin and orientin, another plant-derived compound, displayed synergistic anti-cancer properties when combined in the nanoformulations [57]. Sahab-Negah et al. have designed curcumin-loaded niosome nanoparticles, employing amphiphillic compounds which are able to cross the BBB. This formulation was more effective at inhibiting glioblastoma growth compared to free curcumin in in vitro testing on U87 cells [58,59]. The nanoformulation increased apoptosis and ROS production and showed a decrease in tumor progression markers in vitro., including NF-kB, STAT3 and inflammatory cytokines. These findings are interesting; however, in vivo research is needed before any potential clinical application [59,60].

**Figure 2:**
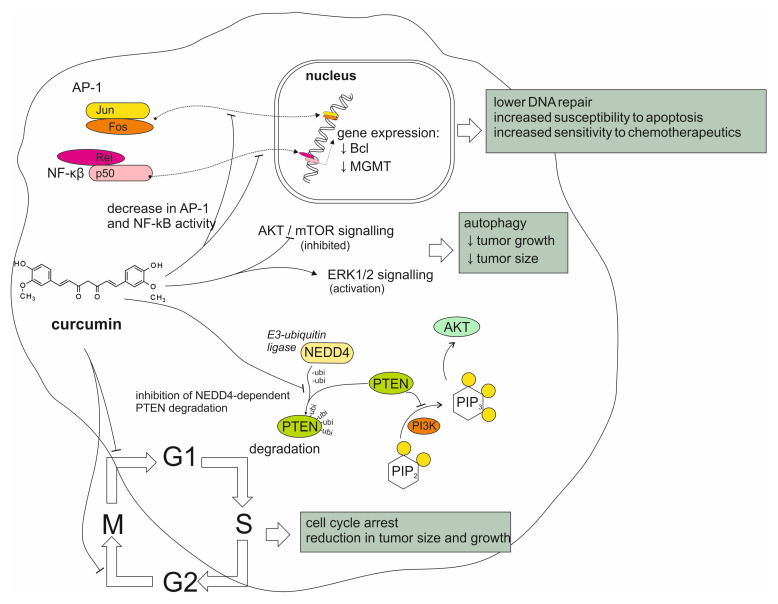
Impact and mechanisms of curcumin action on biology of glioma cells.

In a recent study, Hasan et al. developed nanostructure hybrid lipid capsules enhanced with reversan, an inhibitor of multidrug-resistance-associated proteins. They found that these formulations were effective in delivering a combination of curcumin and the kinase inhibitor regorafenib to GBM cells, leading to an accelerated anti-tumoral effect [61]. Apart from nanoparticles, other drug delivery methods have been explored. Orbay et al. developed a highly porous microgel which could be loaded with hydrophobic curcumin particles and released the drug effectively, with a marked anti-proliferative effect against GBM cells [62]. It is also worth noting that piperine, a compound found in black pepper, has long been known to increase curcumin’s bioavailability by as much as 2000%, and may be worth exploring to help target brain tumors [63]. Curcumin has been reported to exhibit radio-sensitizing potential, which could be useful in overcoming the resistance of various cancers to radiotherapy. As for its potential effectiveness in CNS tumors, in vitro findings have been inconclusive. Zoi et al. have observed a synergistic effect of curcumin and radiation in inducing cell cycle arrest in glioblastoma cells at certain doses, noting, however, a mildly antagonistic relationship at other doses [64]. It has been found that curcumin was able to enhance the effects of ionizing radiation on glioma cells, increasing radiation-induced apoptosis as well as immunogenic cell death by activating endothelial reticulum (ER) stress [65]. In a recent study by Ghanbari et al., it was observed that curcumin in combination with radiation and hyperthermia was more effective in inducing cell death in glioma spheroid model cells, suggesting a synergistic relationship [66]. However, Sminia et al. found that curcumin did not increase the sensitivity of glioma cells to radiation in a significant manner [67]. Thus, further research is needed to elucidate the relationship between curcumin and radiation to determine whether the compound could potentially be used as an adjuvant in glioma radiotherapy. The modes of curcumin action on glioma cells are summarized in Figure 2.

### 4.2. Reports from Animal Models on Curcumin Activity

Curcumin has been shown to block the formation of brain tumors in vivo, with the earliest reported example being brain melanoma in mice, suggesting that intravenous curcumin is able to reach brain tissue [68]. Dietary curcumin has been found to be effective against intracranial gliomas in mice, reducing tumor growth as well as the migration of glioma cells to the contralateral side of the brain (midline crossing) [69]. Rats implanted with GBM cells and treated with intraperitoneal curcumin were less likely to develop tumors compared to the untreated group; in those who did, tumor volume was significantly reduced and apoptosis was increased. Additionally, curcumin did not appear to exert toxic effects as evaluated by standard serum markers (creatinine, alanine transaminase (ALT), aspartate aminotransferase (AST) and others) [47]. The possible use of curcumin as an adjuvant for improving sensitivity to chemotherapeutic drugs for glioma cells has been intensively investigated. Orunoglu et al. found that curcumin-loaded PLGA nanoparticles were most effective in reducing tumor size and growth when administered via intra-tumoral injection, with no significant result when administered intravenously. The authors suggest that modifying the nanoparticles with targeting surface molecules is needed to improve the efficacy of intravenous administration [70]. A dual-drug delivery magnetic system with curcumin and paclitaxel, developed by Cui et al. (as mentioned in the previous section), has yielded satisfactory results in vivo, showing a high level of brain accumulation and an improved survival rate in glioma-bearing mice [55]. In a recent study, Zhuo et al. developed a dual-drug nanoparticulate system, loaded with curcumin and a doxorubicin pro-drug. They found that the formulation was able to effectively cross the BBB and target glioma cells in vivo [49]. Curcumin-loaded nanoliposomes modified with the brain-targeting RDP peptide were shown to cross into the brain and inhibit glioma growth in mice by inducing cell cycle arrest and apoptosis, leading to prolonged survival. The authors suggest that the particles may have been able to enter the glioma cells via endocytosis mediated by acetylcholine receptors [71]. Wang et al. combined curcumin with the topoisomerase inhibitor camptothecin in liposomal nanoformulations modified with tryptamine. They found that the formulation was effective in enhancing the delivery of the two drugs across the BBB and into glioma cells, additionally noting a synergistic effect of curcumin and tryptamine in reducing immunosuppression mediated by regulatory T cells [72]. Exosomes have been explored as another possible drug carrier for curcumin. Jia et al. developed exosomes loaded with curcumin and superparamagnetic nanoparticles, which were used to facilitate magnetic flow hyperthermia. These formulations were effective in crossing the BBB and exerting an anti-tumoral effect on glioma in mice [73]. The potential use of curcumin as a radio-sensitizing agent has also been explored in vivo. In agreement with their in vitro study, Xiu et al. found that combined treatment with curcumin and radiation resulted in stronger anti-tumoral immune responses in CD4+ and CD8+ T lymphocytes as well as CD111c+ dendritic cells, which caused increased levels of immunogenic cell death in glioma cells in mice compared to radiation treatment alone [65]. In a systematic review and meta-analysis of curcumin in glioblastoma in animal models, Luís et al. note that many publications in this subject, though hopeful, do present discrepancies arising from differences in study method (different cell lines, dosage and duration, curcumin formulation, to name a few). The authors also identified that publication bias may skew the body of research towards studies with positive effects. Although this does not discredit the results of these studies or the beneficial effects of curcumin in glioblastoma, it is presently difficult to gauge its exact efficacy in the treatment of this tumor. Additional research, employing the same materials and methods as the existing publications, is needed to further confirm their reliability and identify the details of effective administration before the effective translation of these methods to human clinical trials [74]. The results of studies investigating the effect of curcumin on gliomas in vivo studies are summarized in Table 2.

### 4.3. Reports from Studies Conducted on Humans

Although the anti-tumoral properties of curcumin have been explored in several types of malignancies, few clinical trials have been conducted to investigate the compound’s effects on patients suffering from tumors of the CNS. In a 2016 study by Dützmann et al., thirteen glioblastoma patients were given oral curcuminoids for four days prior to tumor resection. The drug mixture used was a combination of micellar formulations of curcumin and two derivate compounds (demethoxycurcumin and bis-demethoxycurcumin) [18]. The authors detected curcumin in the tissue of the tumor itself, although the concentrations were deemed too low to exert any significant anti-tumoral effects. An increased level of inorganic phosphate in the tumor has been suggested to be a possible result of mitochondrial dysfunction. It was noted, however, that the formulation had a bitter taste, which led three patients to discontinue the study due to nausea, which the authors suggest could be mitigated if capsules were to be used [18]. The lack of therapeutic effect, despite the presence of curcumin in the brain, raises concerns about the low efficacy of the oral administration of this drug. However, the limitations of this study, including its small sample size, make it difficult to draw categorical conclusions. Further research is necessary to find whether curcumin is a worthwhile therapeutic agent and how to utilize it effectively and reliably. A registered ongoing phase I/II study, aiming to determine the efficacy and safety of a combined treatment, is still in the recruiting stage. It is meant to evaluate a therapeutic strategy involving liposomal formulations of curcumin in conjunction with temozolomide and radiotherapy. The study is projected to be completed in 2026–2027 [17,75]. Results from studies investigating the effect of curcumin on gliomas in clinical trials are summarized in Table 3.

## 5. Conclusions

Resveratrol and curcumin are natural ingredients in the human diet and can be easily found in numerous food sources of plant origin. However, before they may be considered as potential additions to standard therapy methods, many issues need to be solved. The chief problem seems to be the matter of bioavailability. Efficient strategies for polyphenol administration and delivery to target brain tissue should be developed. The possible utilization of nanoparticles to improve BBB crossing and finding ways to increase the transport of phytochemicals directly to neoplasmic cells are of the utmost importance. Additionally, it is advisable to further investigate the potential interactions between polyphenols and compounds used in established therapeutic regimens to avoid unfavorable interactions and at the same time possibly exploit mutual complementation.

## Figures and Tables

**Table 1:** Summarized results from in vivo studies investigating use of resveratrol in treatment of gliomas.

Dosage	Animal Species	Tumor Type	Way of Administration	Treatment Period	Results	Ref.
8 mg/kg	Wistar rats	Glioma (C6 cell line)	Oral administration (1 time/day)	Until death of animal	↑ survival time ↑ apoptosis of tumor cells ↑ tumor cells expression of EGFR, MMP-9, NF-κβ, PCNA and COX-2 ↓ tumor cells expression of VEGF and GFAP	[25]
100 mg/kg	Fischer-344 rats	Glioma (RT-2 cell line)	Intraperitoneal injection (1 time/day)	Until 28 days of treatment or animal death	↑ survival time	[37]
0.1 mg/mL (water solution concentration) 50 mg/kg (oral gavage) 5 mg (intra-tumoral injection)	Female BALB/c nude mice	Glioma (U87 cell line)	Water solution of resveratrol served ad libitum Administration through oral gavage (1 time/day) Intra-tumor injections (1 time/day)	Oral administration for 18 days (water or food) Intra-tumor injections for 14 days	In all groups: ↓ tumor volume ↑ apoptosis of tumor cells	[36]
50 μL of 500 μM solution	Sprague-Dawley rats	Glioma (RG-2 cell line)	Lumbar puncture (3 times/day)	3 days	↑ expression of LC-3 and Beclin-1 in tumor cells and surrounding tissues ↑ apoptosis of tumor cells	[35]
5 mg/kg (nanoencapsulated form)	Wistar rats	Glioma (C6 cell line)	Oral administration (1 time/day)	10 days	↓ tumor volume	[38]
15 mg/kg (transferrin (Tf)-modified PEG-PLA nanoparticles conjugated with resveratrol)	Male Sprague-Dawley rats	Glioma (C6 cell line)	Intraperitoneal injection (1 time every 2 days)	Continuous until animal death	↑ survival time ↓ tumor volume	[39]
10 mg/kg (encapsulated in Tf-targeted liposomes)	Female athymic NCr-nu/nu nude mice	Glioblastoma (U87MG cell line)	Intravenous injection (1 time every 3 days)	30 days	↑ survival time ↓ tumor volume	[40]
12.5 mg/kg	BALB/c nu/nu female mice	Glioblastoma (U87MG cell line)	Intraperitoneal injection (1 time a day)	12 days	↓ tumor volume ↑ cytotoxicity of TMZ ↑ TMZ-mediated downregulation of ERK and LC3-II activity ↑ TMZ-mediated cleavage of PARP	[44]
10 mg/kg (conjugated with mPEG–PCL copolymers)	Nude mice	Glioblastoma (U87MG cell line)	Intraperitoneal injection (1 time/day)	14 days	↑ TMZ-mediated anti-tumor effect when compared with control groups	[45]

**Table 2:** Summarized results from in vivo studies investigating use of curcumin in treatment of gliomas.

Dosage	Animal Species	Tumor Type	Way of Administration	Time Period	Results	Ref.
Western-type mouse chow enriched with 0.05% curcumin	C6B3F1 mice	Glioma (Tu-2449 or Tu-9648 cell line)	Oral administration ad libitum	The administration of curcumin was started 7 days before the implantation of malignant cells and continued for 65 or 80 days or until the animal started showing symptoms of tumor presence.	Tu-2449 group: ↑ survival time ↓ tumor size ↓ STAT3 activity Tu-9648 group: ↑ survival time ↓ tumor cell migration to contralateral side of brain ↓ STAT3 activity	[69]
50 mg/kg	Wistar rats	Glioma (C6 cell line)	Intraperitoneal injection (1 time/day)	The administration of curcumin was continued for 10 days.	↓ tumor volume ↓ tumor development	[47]
25 μM (encapsulated in PLGA nanoparticles)	Wistar rats	Glioma (RG2 cell line)	Intra-tumoral injection (1 time/day)	The administration of curcumin was continued until the animals started expressing clear symptoms of significant tumor advancement.	↓ tumor volume	[70]
4 mg/kg (encapsulated in PLGA modified with T7 transferrin receptor ligands and magnetic nanoparticles)	Male BALB/C nude mice	Glioblastoma (U87 cell line)	Intravenous injection (1 time every 3 days)	The administration of curcumin was started 15 days after the implantation of malignant cells and continued for 20 days.	↑ paclitaxel-mediated reduction in tumor volume ↑ paclitaxel-mediated reduction in tumor development ↓ weight loss rate	[55]
20 mg/kg (encapsulated in nanoliposomes modified with RDP peptide)	Male BALB/c nude mice	Glioma (U251MG cell line)	Intravenous injection (1 time every 2 days)	The administration of curcumin was continued for 7 days.	↓ tumor volume ↓ tumor cell proliferation rate ↑ survival time ↓ symptoms severity	[71]
800 μg (encapsulated in exosomes modified with neuropilin-1-targeted ligand—RGE)	Female BALB/c nude mice	Glioma (U251MG cell line)	Intravenous injection (1 time every 2 days)	The administration of curcumin was continued for 28 days.	↓ tumor volume	[73]
30 μM (concentration of curcumin in glioma cells breeding medium)	Male C57BL/6J mice	Glioma (GL261 cell line)	Subcutaneous injection of glioma cells previously treated with curcumin (vaccination) 7 days after initial vaccination, animals inoculated with live glioma cells not previously treated with polyphenol	-	↑ tumor rejection rate	[65]

**Table 3:** Results from clinical trials investigating use of curcumin in treatment of gliomas.

Aim of the Study	Intervention:	Study Design	Results	Ref.
The determination of intra-tumoral and serum levels of curcumin and related curcuminoids that are possible to achieve after the administration of a liquid micellar formulation. Assessment of its clinical tolerance in GBM patients, and the impact of highly bioavailable curcumin on metabolism and energy status in GBM.	1 g of a micellar curcumin formulation (57.4 mg curcumin, 11.2 mg demethoxycurcumin and 1.4 mg bisdemethoxycurcumin) mixed in 200 mL of pear-juice beverage administered three times a day after regular meals for the 4 days before surgery (12 g in total, corresponding to 840 mg curcuminoids).	Ten patients (four women, six men) scheduled for surgery, with histologically proven GBM, a mean age of 67 ± 8 years and a mean body mass index of 26.8 ± 1.8 kg/m^2^. Before and after the curcumin intervention, a 31P MRSI was performed to detect curcumin through changes in metabolites. The MRSIs of the brain were performed on a 3-T whole-body system and planned on T2-weighted images in three orientations. Tissue and blood samples were acquired and flash-frozen during surgery.	The mean intra-tumoral concentrations of curcuminoids among patients who completed the study were as follows: curcumin—56 pg/mg tissue; demethoxycurcumin—8.6 pg/mg; bisdemethoxycurcumin—0.866 pg/mg. The concentrations of curcumin within the tumor tissues correlated with the cumulative received dose. The mean serum concentration of total curcumin was 253 ng/mL. Curcumin admission significantly increased levels of intra-tumoral inorganic phosphate. Intra-tumoral pH increased from 7.06 ± 0.05 to 7.10 ± 0.05, but this result was not statistically significant.	[18]
To assess the safety, tolerability and efficacy of liposomal curcumin in combination with radiotherapy and TMZ in patients with high-grade glioma.	The patients treated with standard chemoradiation (radiation therapy + temozolomide treatment) will receive liposomal curcumin at doses of 300–400 mg/m^2^ weekly.	A phase I/II multi-center, open-label, dose-escalation study in patients with high-grade malignant gliomas. Dose finding will be performed using a time-to-event Bayesian optimal interval (TITE-BOIN) rule-based schema. Approximately 50 patients will be screened to achieve up to 30 patients assigned to the study’s intervention. The duration of treatment for each patient will be up to 34 weeks. Treatment starts with the beginning of infusion and ends, if tolerated, at the end of cycle 6 of adjuvant TMZ. The dose-limiting toxicity evaluation period is 10 weeks. Afterward, the patients will continue adjuvant TMZ and liposomal curcumin for an additional 24 weeks. Progression assessments accomplished with MRI evaluations are planned for the 10th, 18th and 26th weeks of treatment. Patients will be monitored for survival and progression for the first 2 years after the last dose of curcumin every 2 months. After the first 2 years, patients will be monitored every 6 months.	The study is currently in the recruitment phase. So far, no dose-limiting toxicity has been observed; the maximal tolerated dose of curcumin was assessed to 300 mg/m^2^. The research is projected to be completed in 2026–2027.	[17,75,76]
